# Determining the Requirements and Barriers for Using Barcode Technology in the Hospitals of Tabriz, Iran

**Published:** 2017-05

**Authors:** Peyman REZAEI-HACHESU, Leila ZYAEI, Hadi HASSANKHANI

**Affiliations:** 1. Dept. of Health Information Technology, School of Health Management and Medical Informatics, Tabriz University of Medical Sciences, Tabriz, Iran; 2. Faculty of Nursing and Midwifery, Tabriz University of Medical Sciences, Tabriz, Iran

## Dear Editor-in-Chief

Medical Errors are one the major concerns of on-site health care (1). These errors can reduce with the automatic identification technologies such as barcode (2, 3). Barcode technology can prevent medical errors by providing detailed and reliable information to the site of patient care (4, 5). The barcode is one of the most powerful and economical methods of improving the patient safety. While many health care organizations do not tend to apply the barcode system due to lack of proper understanding through the technology and its system requirements (6).

Considering the importance of effective using the barcodes and removing the barrier of using this technology, this study aimed to examine the requirements and barriers for using the barcode technology in hospitals affiliated to Tabriz University of Medical Sciences between 2013 and 2014. This is an observational and descriptive study performed in 10 teaching hospitals. The primary checklist was designed by the researchers in order to collect data by studying scientific sources (3, 6–10) and regarding three factors including organization, technology, and budget. The final tool was set in the form of two checklists including the requirements with 26 components and barrier with 13 components. The selection basis of personnel was relevance of their duties to the most important processes of hospitals requiring the use of barcode technology. The data gathered from 65 participants. Ninety percent of assessed hospitals did not evaluate the required changes in the workflow processes of hospital. Although the property of the barcode symbols was evaluated in 40% of hospitals, however, there was no knowledge of international standards for barcodes in none of the hospitals. Only 20% of hospitals considered the issues related to staffing for working with the barcodes such as the cost and training. Moreover, despite the importance of integrating barcode system with hospital information systems (HIS) for the applicability and effectiveness of barcode technology, this integration was not found in 80% of hospitals.

**Fig. 1:**
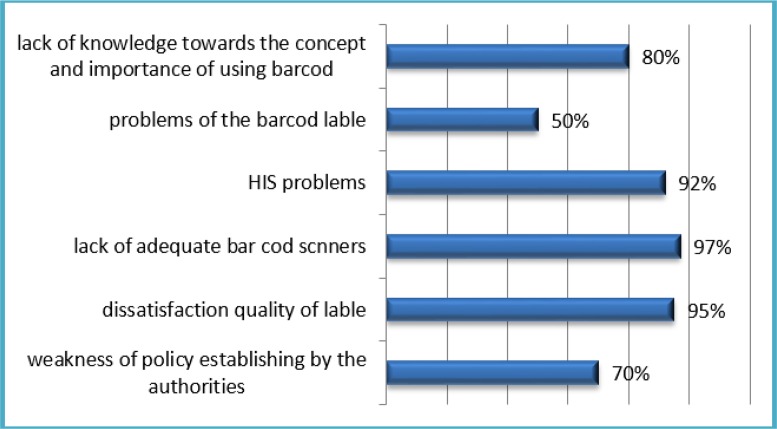
Barriers from the perspective of employees after the implementation of the barcode

The barriers for using the barcodes were studied in two aspects. First, the barriers which hospitals may deal with prior to the implementation of barcodes; and the second, the difficulties and barriers that could have arisen after the adoption of barcodes and can stop using them. Fifty Percent studied hospitals had not the using of barcode policy. However, in the other 50% of hospitals, there was the use of barcode policy, but neglect, poor attention, and also the absence or weakness of identified and detailed planning in the process of implementing prevents its application. The lack of qualified personnel for implementation and lack of integration of bar codes with the HIS system in the radiology and laboratory section was observed in the half of the hospitals. In the 80% of the studied hospitals, there was not understanding of the requirements for implementation. Noteworthy 70% of participants believed that lack or weakness of policy establishing by the authorities along with the neglect of the hospital management prevents the planning for application of the barcodes (Fig. 1).

Additionally, 80% of them stated that lack of knowledge towards the concept and importance of using barcodes cause to negligence of applying this technology.

Staff training and resistance due to the weakness sense for adoption of the assigned duties has been mentioned as one of the main barriers for application of barcode process (3).

The requirements and barriers can be classified into the three major groups of organization policy and processes issues, technology, and education and costs. Lack of approving or notifying a specific policy for the use of barcodes by organizations such as the Ministry of Health and lack of knowledge, understanding toward the infrastructure, and inadequate staff training are the greatest barriers to the successful implementation of bar code system.
